# Retraction Note: Study of defensive behavior of a venomous snake as a new approach to understand snakebite

**DOI:** 10.1038/s41598-025-88686-x

**Published:** 2025-02-11

**Authors:** João Miguel Alves-Nunes, Adriano Fellone, Selma Maria Almeida-Santos, Carlos Roberto de Medeiros, Ivan Sazima, Otavio Augusto Vuolo Marques

**Affiliations:** 1 Programa de Pós-Graduação em Biodiversidade, Instituto de Biociências, Letras e Ciências Exatas, Universidade Estadual Paulista “Júlio de Mesquita Filho”, São José do Rio Preto, São Paulo Brazil; 2 Laboratório de Ecologia e Evolução, Instituto Butantan, São Paulo, São Paulo Brazil; 3 Programa de Pós- Graduação em Ecologia e Evolução, Universidade Federal de São Paulo, Campus Diadema, São Paulo, Brazil; 4 Museu de Biodiversidade Biológica, Instituto de Biologia, Universidade Estadual de Campinas, Campinas, São Paulo Brazil

Retraction of: *Scientific Reports* 10.1038/s41598-024-59416-6, published online 03 May 2024

The Editors have retracted this Article.

After publication, concerns were raised about the animal work reported in this paper. The Animal Ethics Committee of Butantan Institute confirmed that the approval granted to the Authors for their research on snakes (Bothrops jararaca) did not include newborn snakes or the use of the “soft stepping” method.

All authors disagree with this retraction.

